# Manual therapy for chronic migraine: a pragmatic randomised controlled trial study protocol

**DOI:** 10.1186/s12998-019-0232-4

**Published:** 2019-03-27

**Authors:** Jim Odell, Carol Clark, Adrian Hunnisett, Osman Hassan Ahmed, Jonathan Branney

**Affiliations:** 1 Bournemouth University, Royal London House, Christchurch Road, Bournemouth, BH1 3LT UK; 2 BPP University, McTimoney College, Kimber Road, Abingdon, OX14 1BZ UK

**Keywords:** Chiropractic, Chronic migraine, Headache, Manual therapy, Allodynia, Randomised controlled trial

## Abstract

**Introduction:**

Chronic migraine is a largely refractory condition affecting between 1 and 2.2% of the overall population worldwide, with females more affected than males. There are also high health and socioeconomic costs associated both for the individual and society. The mainstay of chronic migraine management is pharmacological, but the options available have limited efficacy and there are often unwanted side effects. There is some evidence for manual therapy as a treatment option for migraine, but its effectiveness for chronic migraine is unknown. Therefore, we have designed a pragmatic randomised control trial to investigate whether adding manual therapy to the tertiary specialist treatment of chronic migraine improves patient-reported outcomes.

**Methods:**

A pragmatic, randomised controlled trial in a hospital tertiary headache clinic**.** Participants will be randomised into one of two groups: treatment as usual or treatment as usual plus manual therapy. The primary outcome measure will be a change in the Headache Impact Test score. Secondary outcomes will also be measured over the 12-week study period including changes in headache frequency, migraine specific quality of life and reductions in relevant medicine consumption. The manual therapy group will have five treatment sessions each lasting 30 min. The recruitment target of 64 participants will allow power at 80% with *p* = 0.05 using minimal clinical difference for Headache Impact Test of 3.7 and includes provision for a 10% dropout rate. Recruitment will take place between August 2018 and February 2019. The results will form part of a doctoral study and be published in peer-reviewed journals and presented at national/international conferences.

**Discussion:**

Current pharmacological approaches have limited effects in the management of chronic migraine and there is a requirement to improve treatment options and reduce the health and economic burden of the condition. Manual therapy has been shown to be effective in other chronic pain conditions as well as other primary headaches. This study will explore the effectiveness of manual therapy as an adjunctive approach to the management of chronic migraine.

**Trial registration:**

The trial has received a favourable opinion from the UK Health Research Authority (IRAS 228901) and is registered at ClinicalTrials.gov.number NCT03395457. Registered 1st March 2018.

## Background

Migraine is experienced by the vast majority of sufferers as episodic migraine (EM) [1, 2] occurring regularly although not necessarily frequently, with around 65% of sufferers estimated to have a migraine episode fortnightly to monthly [3]. However if the episodic pattern becomes uncontrolled, a process of chronification occurs whereby the original episodic migraine becomes very frequent and more disabling. This is termed chronic migraine (CM) and is described by the International Headache Society (IHS) classifications [4] as “*headache occurring on 15 or more days per month for more than three months, which, on at least 8 days per month, has the features of migraine headache*”. On the days without migraine headache the individual can often suffer from pre and post headache effects adding to the burden of this condition.

The management of CM is more complex than EM, as it is often resistant to standard treatments with resultant additional costs compared to EM. The mean (SD) annual cost per CM person ($8243 [$10,646]) was over three times that of those with EM ($2649 [$4634]). Both direct medical costs and cost of lost productivity were substantially higher in CM than EM [5]. In addition to financial costs, the social costs and personal impact of CM often leads to severe disability for those with CM [6, 7]. This is especially true for females, with the annual prevalence of CM in women being 1.7–4.0% compared to men (0.6–0.7%) [8–11]. Those women aged between 18 and 49 years of age are the most affected across the range of measures. In one study, CM sufferers were found to be three times more likely to have lost work and have reduced household productivity than those with EM (58% compared to 18%) [12]. People with CM are also much more likely to report “very severe headache-related disability” as measured by the Migraine Disability Assessment Scale (MIDAS) than those with episodic (24.8 and 3.2% respectively) [13, 14].

Despite the suggestion that migraine is a syndrome with multiple pathological mechanisms which support a multi therapeutic approach rather than a single approach [15–17], the mainstay of treatment for CM is pharmacological. However, some patients do not want, or cannot, take some prophylactic medications such as Topiramate due to restrictions in its use [18]. Currently, OnabotulinumtoxinA (Botox) is the only specifically licensed treatment for CM in the UK [19]. Although its mechanism of action is unclear, studies have demonstrated that injecting specific sites on the head and neck produce significantly beneficial effects in CM patients. One study concluded that 32% of CM patients achieved a 50% reduction in headache days and a 50% reduction in migraine days, with NICE guidance recommending a 30% reduction in headache days as a measure of success in Botox studies [20]. Although similar clinically significant reductions in the Headache Impact Test (HIT 6) scores are seen with Botox and Topiramate interventions, Botox has fewer side effects and higher adherence rates [21, 22]. However, the efficacy of Botox in those patients who benefit only partially or not at all from Botox, and its relatively high cost, are barriers to an increased uptake [23–25]. Therefore if adjunctive therapies, especially those with fewer side effects and relatively low costs could be utilised, this may increase the benefit to more of those with CM.

One possible adjunctive intervention is manual therapy. Despite the mechanisms of its potential action in CM being unknown, the basis for its potential role can be garnered from studies in associated conditions. These include primary headaches (tension type and migraine), as well as common comorbidities; chronic pain and specifically neck pain. One systematic review concluded that MT has an efficacy in the treatment of chronic tension headache equal to that of prophylactic medication with tricyclic antidepressant [26]. Another involving massage therapy and chiropractic spinal manipulative therapy concluded that they may be as efficient as Propranolol and Topiramate in migraine prophylaxis. It also concluded that there is moderate quality evidence for both spinal manipulation and mobilisation as suitable treatments in chronic non-specific neck pain [27]. These studies found benefits in at least one of the following: frequency, duration and intensity of headaches/pain.

Currently there are two theories in existence relevant to this study of manual therapy in the management of chronic migraine. One is that migraine is, in part, an abnormal response in those genetically pre-disposed, to nociceptive input involving the nerves of the upper cervical spine (C1, C2 and C3) and associated joints and muscles. This leads to exaggerated sensitisation of the trigeminal pathway and subsequent face, neck and head pain [28–30].

The other theory incorporates the allostatic model in which people respond to stressful events, actual or perceived, with physiological and behavioural changes. In general these changes maintain normal physiological stability (allostasis). However, if stressors (including ongoing pain or nociceptive inputs) become too great or frequent then the normal response can become dysfunctional as a result of allostatic loading, which itself alters brain structure and function. Likewise, repeated migraines are themselves thought to be a driver of changes to the brain structure that may lead to a dysfunctional allostatic response. Consequently, many migraine sufferers report that stressful activities of daily living (physical and emotional) exacerbate their migraines [31, 32].

Chronification from EM to CM may be a result of the stress mechanisms generating the migraine or as a consequence of changes in the brain arising in response to the attacks. Therefore if stressors (for example nociceptive pain, and central sensitisation, possibly from musculoskeletal disorders) can be reduced, this may add to the efficacy of existing interventions [33].

Whilst the process of chronification in CM is not fully understood, there are a number of known associated risk factors including head and neck injury, and other pain disorders [34] (Table 1).

**Table 1:** Risk factors associated with migraine chronification and reversion

• Obesity	
• Snoring	
• Sleep disorders	
• Excessive caffeine intake	
• Psychiatric/psychological disease (Depression/Anxiety)	
• High baseline headache frequency	
• Overuse of migraine abortive drugs	
• Major life changes	
• Head or neck injury	
• Cutaneous allodynia	
• Female sex	
• Comorbid pain disorders	
• Lower socioeconomic status	

(Adapted from Schwedt, 2014, [79])

Currently, one of the most common factors in chronification is considered to be the presence of medication over use headaches (MoH) due to the frequent use of opioids and barbituates in self medication of headaches, or in association with concomitant conditions. Estimates of between 30 and 72% of CM patients presenting at tertiary clinics are thought to have MoH associated chronification [35, 36]. The IHS diagnosis of medication overuse is made if abortive drugs are used regularly for more than three months on 10 or more, or 15 or more, days a month, depending on the drug. One study estimated a twofold increase in chronification with opioid use on 8 days a month [37]. Therefore any studies of interventions in CM should include a detoxification phase or a process to mitigate CM with MoH being included.

Both of the above migraine models suggest a role for altered sensory processing in the brainstem, which is associated with the presence of central sensitisation and one of the consequences, cutaneous allodynia (CA). Some studies cite CA as more prevalent in CM than EM, whilst others suggest there is little difference and it is more a reflection of migraine duration. There are a few potential reasons for these differences which include: the type of CA – thermal, dynamic and static mechanical; how it is measured and the temporal nature of CA making it difficult to measure consistently; and its role as a marker for the risk of frequent attacks rather than simply a consequence of frequent attacks [38, 39]. CA is also consistently reported more in females than males (49.7% vs 32.6%%, *P* < 0.001) [40] and is common in other chronic pain conditions, with its presence associated with a reduction in the efficacy of abortive treatments [41, 42].

Various models of MT and pain reduction exist which involve biomechanical, neurophysiological and psychological components, either individually or in combination. However all of these models consider that MT may work by activating descending inhibitory pathways via different levels of the spinal cord [43–45]. The common relationship of central sensitisation, the cervical spine, and pain disorders (see Table 1) would suggest MT may have utility in the management of CM.

In terms of potential as an adjunctive treatment, MT has been shown to reduce pain and have a direct effect on the mechanics of the cervical spine that results in functional improvements [46–48]. MT has also been shown to increase local pain pressure thresholds, which are used to detect central sensitisation [49, 50]. Consequently, MT may reduce cutaneous allodynia and improve the efficacy of current approaches to treating CM.

The above discussion outlines commonalities between migraine, other pain conditions, its chronification and the potential for MT in its treatment. Combined with evidence showing the scope for improvement in existing CM treatment, this suggests there may be potential for the use of adjunctive non-pharmacological treatments, especially if these are generally associated with lesser side effects [16, 51].

The objective of this study is to determine the effectiveness of MT as an adjunctive therapy to tertiary care (‘care as usual)’ in CM.

## Methods

### Study design

**Fig. 1:**
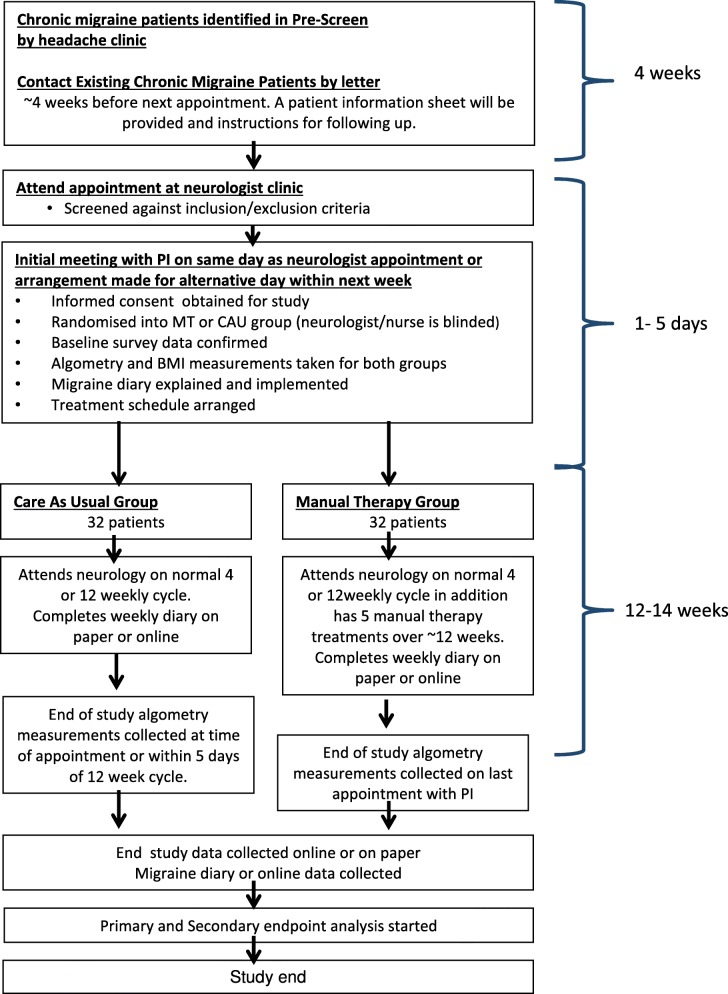
Participant flow

This will be a single centre, pragmatic Randomised Controlled Trial (RCT) involving 64 participants. The two groups will be “Tertiary care as usual” and “Tertiary care as usual plus MT”. The objective of the study is to measure the difference between the two treatment groups in CM. The study design adheres to the IHS and Consolidated Standards of Reporting Trials (CONSORT) guidelines [52, 53]. The study flow is outlined in Fig. 1.

### Participants

#### Recruitment

Participants will be recruited between August 2018 and February 2019 through the Salford Royal NHS Foundation Trust acute neurology clinic service in the UK. The neurologist and specialist headache nurse will identify potential participants from their list of existing CM patients.

Details of this study will be sent to potential participants with their clinic appointment letter. Eligible participants will be female aged greater than 18 years of age, and diagnosed with CM according to the criteria of the International Classification of Headache Disorders (ICHD-III) by their neurologist. The enrolment in to tertiary care requires that patients must be free of medication over use headache and as part of the inclusion criteria (Table 2), participants will have gone through a detoxification phase and had at least one cycle of tertiary care treatment to further mitigate medication overuse headache. Exclusion criteria for this study are: contraindication to MT including spinal radiculopathy; uncontrolled psychological conditions; and any physical or MT in the last 6 weeks. Acute and abortive migraine medication will continue as usual during the study.

**Table 2:** Inclusion and exclusion criteria

Inclusion Criteria	
• Females over 18 years of age	
• A good command of English (to enable informed consent)	
• Existing patients with chronic migraine as diagnosed by a clinical interview with a neurologist in line with the International Classification of Headache Diagnosis criteria (ICHD)	
• Undergoing care as usual from the neurologist	
• Must have had at least one cycle of treatment from neurologist and not be a new patient	
Exclusion Criteria	
• Currently having or had manual therapy to the neck or shoulder in the previous six weeks.	
• A new patient without any existing management by neurologist	
• Having a condition contraindicated for manual therapy including, but not limited to, inflammatory disorders, severe osteoporosis and tumours.	
• Identification of any medical ‘red flags’ by the neurologist.	
• Evidence of any central nervous system involvement for example:	
o Facial palsy (presence of ptosis/Horner’s syndrome)	
o Visual disturbance (presence of blurred vision, diplopia, hemianopia)	
o Speech disturbance (presence of dysarthria, dysphonia, dysphasia such as expressive or receptive)	
o Balance disturbance (presence of dizziness, imbalance, unsteadiness, falls)	
o Paraesthesia (presence, location such as upper limb/lower limb, face)	
o Weakness (presence, location such as upper limb/lower limb)	
o Known major psychiatric or psychological conditions not under control	

### Inclusion and exclusion criteria

#### Sample size

The study is powered at 80% (α =0.05) to detect a mean difference of 3.7 points in HIT6 before and after treatment. A pooled calculation of standard deviation (SD) of 4.91 was calculated for the Headache Impact Test (HIT6) from the analysis of major studies in chronic migraine [7, 54–59]. Although this requires 29 participants in each group, 32 will be recruited to allow for attrition that may be experienced over a 12 week period.

#### Allocation

A randomisation assignment sequence will be created by an independent member of the research team using randomisation software “Research Randomizer” [60]. The results will be put into sealed opaque envelopes which the participants will open in their meeting with the PI, who will explain the process to be followed depending on the group allocation.

Immediately following the initial screening by the neurologist/specialist nurse, those participants who meet the inclusion criteria will be invited to an assessment with the Principal Investigator (PI). If this is not possible, it will be arranged within 5 days. The PI will provide participants with verbal and written information about the project before gaining written consent. He will also explain the risks, and benefits as well as the potential adverse reactions to MT and how to manage them. Potential adverse reactions may include temporary local tenderness, aching and tiredness [61]. Participants will be randomised into two groups (care as usual and care as usual plus MT).

The neurologist will be blinded as to the participant’s group allocation in order to reduce initial selection and potential contact bias. The PI cannot be blind to the manual therapy group but will be blinded to the end of study survey analysis, with an independent member of the research team detailed to recode the participant reference numbers and store the key file on a password protected computer. The unblinding of single cases by the PI/CI in the course of a clinical trial will only be performed if necessary for the safety of the trial subject.

#### Intervention

The neurologist will manage the care as usual group alone. The care as usual and MT group will be managed by the neurologist and receive MT from the PI. The MT administered will consist solely of mobilisation, manipulation and soft tissue work. The PI will use a pragmatic approach to the MT administered, using any of the above manual interventions deemed clinically appropriate at each appointment based on assessment prior to treatment and participant feedback. The PI is an experienced chiropractor with over ten years’ clinical experience of chiropractic and soft tissue work, with postgraduate education and training in the management of CM. The treatment delivered will be recorded in the Case Report File (CRF). Failure to attend all manual therapy sessions will result in withdrawal from the study although provision will be made to provide an alternative appointment if the participant cannot make one as planned. Any change in a participant’s medical condition such that they fulfil the exclusion criteria above will result in their withdrawal from the study. Data from these participants would not be included in the final study results. Adverse events will be recorded in the CRF, and if classed as serious would be referred to the neurologist to be reported to the sponsor within 24 h of learning of the event and to the Main Regional Ethics Committee within 15 days in line with the required timeframe. The outline interventional procedure is shown in Table 3 with the project flow following the SPIRIT guidelines (Table 4).

**Table 3:** Manual therapy protocol

1. Assess upper body^a^ posture in sitting	
2. Assess active and passive neck range of motion	
3. Assess shoulder girdle range of motion by raising each arm sideways from side of body up to ear	
4. Assess the temporomandibular joint	
5. Identify areas to treat in sitting position	
6. Administer MT using mobilisation, manipulation and soft tissue release in sitting position	
7. Assess patient shoulder girdle, neck and head supine and prone	
8. Administer MT in supine and prone position	
9. Following each session an outline of the MT used will be recorded.	
10. A total of 30 min will be allocated for each participant at these consultations	

^a^Upper body defined as from thoraco-lumbar junction upwards

**Table 4:**
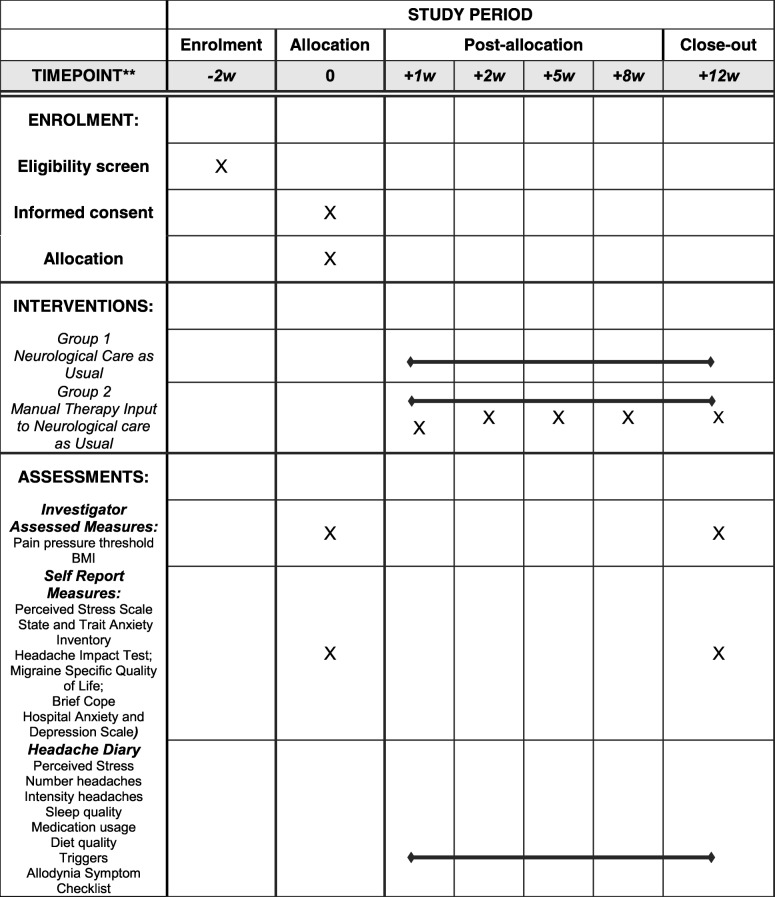
Project Flow Manual Therapy for Chronic Migraine

#### Outcome measures

The primary and secondary endpoints are differences in within-group changes between groups in the outcomes listed below (Table 5). These will be measured using the weekly diaries and the validated instruments in the baseline and 12-week follow-up questionnaires.

**Table 5:** Outcome Measures

Primary Outcomes	Measurement Instrument (validity)
Migraine-related disability	Headache Impact Test (HIT 6) [54]
Secondary Outcomes	Measurement Instrument
Quality of Life	Migraine Specific Quality of Life Questionnaire (MSQ. V2.1)[59]
Use of abortive migraine medications	1.1.11.1.11.1.1Diary [69]
Percentage of participants with reduction in headache frequency (days per month) of greater than 50%	
Number of headache free days	
Stress	
Stress	Perceived Stress Questionnaire (PSS) [80]
Stress	Brief Cope [81]
Anxiety	State and Trait Anxiety Inventory (STAI-6) [63]
Anxiety & Depression	Hospital Anxiety and Depression Scale (HADS) [82]
Cutaneous Allodynia	Allodynia Symptom Checklist (ASC) [83]
Cutaneous Allodynia	Algometry [49, 84]
Patient Reported Outcome Measure	Patient Global Impression of Change Scale (PGICS) [85]

#### Data collection

All participants will be asked to complete a baseline questionnaire, comprising demographic information and the following validated instruments: Headache Impact Test (HIT6), the Migraine Specific Quality of Life Questionnaire (MSQ.V2),The perceived Stress Scale (PSS-10), State and Trait Anxiety Inventory (STAI-6),Brief Cope, Hospital Anxiety and Depression Scales (HADS) and the 12 Item Allodynia Symptom Checklist (ASC-12) The questionnaire will be available in paper or online format for completion at the initial assessment or at home at the discretion of the participant.

BMI values (from height and weight) will be calculated in the clinic and pain pressure thresholds will be taken using a somedic electronic pressure algometer [62] on each of the following muscles: Frontalis, Trapezius and Subocciptal. The order of these measurements will be taken randomly, which is an approach used in previous migraine studies [47, 48]. A distal site (forearm) will also be included to help provide a measure of wide spread central sensitisation [63]. These measures will be taken again at the end of the study after 12 weeks. Individuals assigned to the tertiary care as usual plus MT group will be given a 12 week treatment plan as recommended in IHS guidelines [52]. Sessions will be planned for Weeks 1, 2, 5, 8 and 12.The care as usual group will complete the normal 12 week cycle with the neurologist. Both groups will complete weekly diaries and an end of study questionnaire that duplicates the baseline questionnaire and includes a Patient Global Impression of Change Scale.

To encourage completion of data the participants will be encouraged to complete paper versions of the questionnaire at the first visit in the waiting area and leave it at reception, rather than taking home. Online versions will also be available. Reminder text message or emails will be sent out each week to encourage completion of the paper or online weekly diary.

#### Data management

The questionnaires and diaries will not contain information that can identify individuals, only an individual reference number. All information used for this research will be kept securely on password protected computers and only accessible by members of the research team. Any data that can identify a participant will be destroyed within 3 months of final data collection. Anonymous data may be kept for up to 5 years on a secure university computer.

### Statistical analysis

Descriptive demographic and clinical characteristics will be collected, using means and SDs for continuous variables and proportions and percentages for nominal variables. Each group will be described separately. Quantitative data collected from each group will be analysed using SPSS ^(^™^)^ Version 24 software. Parametric tests will be used to analyse data wherever it is normally distributed, and non-parametric will be utilised for non-normal data.

The mean and standard deviations of primary and secondary outcomes will be reported, with an analysis of between- group differences over the 12 weeks using Students T test or Mann-Whitney. The effect size will be calculated using the following figures: small (0.2–0.5), medium (0.5–0.8) or large (> 0.8). Repeated measures ANOVA will be used to explore the differences between intermediate time points. If appropriate, multivariate linear regression (MANOVA) will assess the secondary outcome relationship with changes in primary outcomes. Correlation between primary and secondary outcomes will use Pearson’s coefficient.

## Discussion

Two high quality RCTs have explored the effectiveness of MT in those with migraine, of which only one study specifically reported on those with a diagnosis of CM [64, 65]. This was a 3-Armed RCT with 105 participants using osteopathy (MT) and medical therapy, sham and medical therapy and medical therapy alone. The results were a reduction in HIT-6 score by an average of− 8.74; *p* < 0.001 with OMT compared to medication care as usual. There were also statistically significant (*p* < 0.001) reductions in medication use and migraine frequency. In comparison, this study will use only one practitioner instead of six, and five sessions over three months instead of eight sessions over six months. The technique used will be chiropractic rather than osteopathy, although similar soft tissue release and mobilisation techniques will be used (albeit on a broader area of the shoulder girdle and neck). This difference with a crossover of similarity should strengthen the theory that manual therapy of different types can produce similar adjunctive benefits and add to the dose benefit discussion.

There is evidence to suggest that MT as an adjunct to Botox improves outcomes in those with CM. This comes from a pilot study with 22 participants [66]; one group had transcutaneous electrical nerve stimulation (TENS) and Botox, the other MT and Botox. Each group had one 30 min session per week over 4 weeks. Both groups showed a reduction in medication use and pain pressure thresholds in the MT group compared to the TENS. This study aims to build on the outcomes of previous studies focussing on MT as an adjunctive therapy to ‘usual care’ for those receiving treatment in a tertiary care setting. At the time of writing, this study is thought to be the first to measure central sensitisation using algometry in CM whilst tracking changes using the Allodynia Symptom Checklist. This will allow greater understanding of potential temporal changes in central sensitisation with treatment outcomes. It should also provide a comparison between the baseline and final algometry changes and the Allodynia Symptom Checklist measures.

### Methodological considerations

The study is designed to be pragmatic and to balance the needs of efficacy and effectiveness in clinical research [67]. The HIT 6 score is used in this study as the primary outcome measure, as this is the only headache disability measurement tool with established validity in CM [54, 57]. The number of headache/migraine days will be collected as a secondary measure using a headache diary, a well established and valid measurement instrument [68–70] and recommended by the IHS.

Blinding and the placebo effects are potential limitations in this study. Although it is impossible to blind the PI for treatment, the final results will be re-coded to reduce the potential for analysis bias. The placebo effect is known to be high in all headache studies with acute treatment placebo being higher than prophylactic and tablets lower than injection [71, 72]. In studies of botox in chronic migraine, both in the immediate 12 week cycle and in longer term studies over multiple cycles, authors have cited placebo levels of between 20 and 49% [73, 74].

It is recognised that the MT group may experience a higher level of placebo as a result of the necessary interpersonal interactions [75, 76]. However in this study there is a high level of personal interaction with the headache nurse who applies the medical interventions and thus may be considered a balancing to interactions in the MT sessions.

The external validity of the RCT may be a weakened as a result of the involvement of only one manual therapist, and thus could limit the measure of consistency of outcomes across different practitioners. Conversely, this will reduce the risk of potential multiple bias that comes with the use of multiple therapists. To mitigate reductions in external validity, the study focuses on a specific group in a ‘real life setting’ (females aged 18 years and above with CM in tertiary clinics) [77]. It will measure factors including stress, depression, coping styles, anxiety, diet, sleep quality, and allodynia, all of which potentially affect the primary and secondary outcomes. Although the IHS recommend a placebo control for prophylactic pharmacological studies, there is evidence that less than 10% actually conform [78] and likewise despite having two active arms this study also lacks a placebo control group. To address this limitation, and in the absence of IHS guidelines for non-pharmacological trials, the study design will adhere closely to the recommendations for IHS pharmacological RCTs in chronic migraine. The CONSORT guideline for non-pharmacological studies will be followed which provides a systematic approach to ensuring quality and the external and internal validity of clinical studies.

#### Scientific value

CM is a highly disabling condition with few evidence-based treatment options. In the UK, Botox is the only specifically approved pharmacological intervention, although Topiramate and occipital nerve injections are used in tertiary clinics. This study will aim to explore the outcomes of MT as an adjunct to those receiving ‘care as usual’ for CM in a tertiary setting. It will also provide information relating to central sensitisation in the form of CA for participants, which will contribute to a body of knowledge in those with CM and support tailoring treatment options.

Although this will be a pragmatic study, the results of this study will only be applicable to the target group - females over 18 years of age with CM under tertiary care. The approach used will however be generalisable to all manual therapists with the requisite training in techniques used. Undertaking the study in a specialist medical headache clinic should help to build the nascent relationship between medical headache specialists and manual therapists. Post study dissemination will potentially help the development of more multidisciplinary approaches.

## Abbreviations

ASC: Allodynia Symptom Checklist
CA: Cutaneous Allodynia
CM: Chronic Migraine
CRF: Case Report File
EM: Episodic Migraine
HADS: Hospital Anxiety and Depression Scale
HIT6: Headache Impact Test
IHS: International Headache Society
MIDAS: Migraine Disability Assessment Scale
MoH: Medication Overuse Headache
MSQ. V2.1: Migraine Specific Quality of Life Questionnaire
MT: Manual Therapy
PGICS: Patient Global Impression of Change Scale
PI: Principal Investigator
PSS: Perceived Stress Questionnaire
STAI-6: State and Trait Anxiety Inventory
